# Enhancing aortic anastomotic integrity: ex vivo evaluation of reinforcement techniques using Teflon-felt sandwich with mattress sutures

**DOI:** 10.1007/s12055-025-02043-1

**Published:** 2025-08-29

**Authors:** Hironari No, Kenji Iino, Hirofumi Takemura, Chihiro Watanabe

**Affiliations:** 1 Department of Cardiovascular Surgery, Kanazawa University, 13-1 Takaramachi, Kanazawa, 920-8641 Japan; 2 Faculty of Mechanical Engineering, Institute of Science and Engineering, Kanazawa University, Kanazawa, Japan

**Keywords:** Aortic artery anastomosis, Teflon-felt sandwich, Polytetrafluoroethylene, Surgical anastomosis, Suture techniques, Tensile tension test

## Abstract

**Purpose:**

The success of aortic surgery depends on the durability and hemostatic properties of anastomotic techniques to prevent complications such as bleeding, thromboembolic events, and anastomotic disruption. This study evaluated the tensile strength of different suturing techniques ex vivo to assess their suitability for aortic procedures.

**Methods:**

Aortic wall specimens were obtained from porcine ascending aortae and anastomosed to vascular grafts using a 5–0 polypropylene monofilament suture. Four different suturing techniques were evaluated: simple interrupted sutures (Group A, *n* = 14), simple interrupted sutures with adventitial inversion and horizontal mattress sutures (Group B, *n* = 14), simple interrupted sutures with a Teflon-felt sandwich (TFS) (Group C, *n* = 14), and simple interrupted sutures with a TFS combined with horizontal mattress sutures (Group D, *n* = 14). The maximum tensile strength of each anastomosis was assessed.

**Results:**

The simple interrupted suture technique incorporating a TFS with horizontal mattress sutures (Group D) demonstrated significantly superior tensile strength compared with the other techniques (*P* < 0.001). Notably, in this group, the fracture site was near the Teflon horizontal mattress suture, whereas in the other groups, fractures occurred adjacent to the simple interrupted sutures.

**Conclusion:**

The TFS technique demonstrated superior tensile resilience among the studied methods, suggesting its potential advantage in aortic surgery. These findings provide a strong theoretical foundation for optimizing anastomotic techniques and warrant further in vivo investigation to validate their clinical applicability in enhancing surgical outcomes.

**Graphical abstract:**

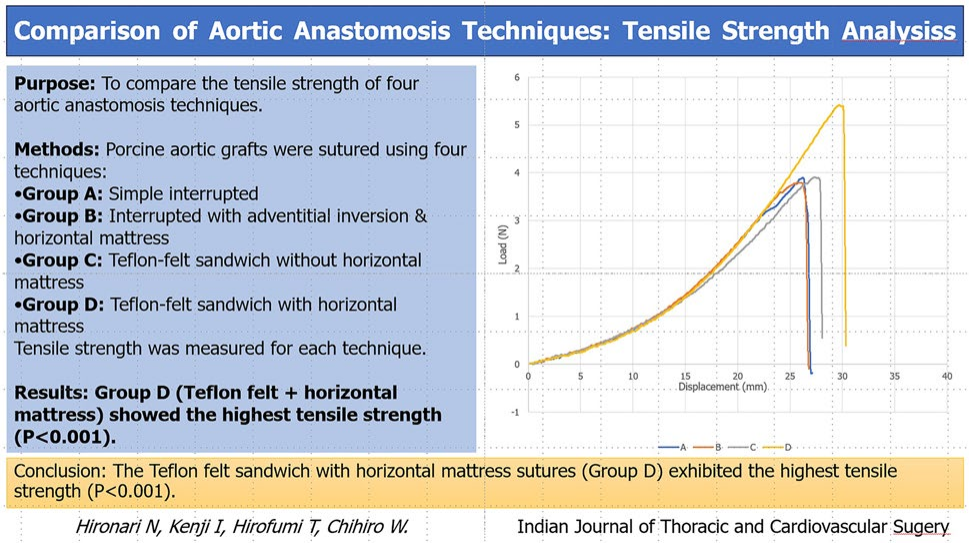

**Supplementary Information:**

The online version contains supplementary material available at 10.1007/s12055-025-02043-1.

## Introduction

Despite advancements in aortic surgery, achieving a secure anastomosis remains a critical technical challenge due to the mechanical properties of the aortic wall. While numerous reinforcement techniques have been introduced in clinical practice, the biomechanical characteristics of these methods have not been sufficiently studied in normal aortic tissue under controlled conditions. The fragile nature of the aortic wall presents challenges when sutured to a vascular graft, frequently resulting in hemostatic issues or the creation of new entry sites. These problems can lead to life-threatening bleeding or require additional surgical interventions. To reduce such risks, various anastomotic and reinforcement methods have been developed to improve the safety and durability of aortic surgery outcomes [1–6]. For example, techniques employing external felt reinforcement or adventitial inversion have been developed to improve the mechanical integrity of the aortic wall. However, their biomechanical performance has not been thoroughly compared under standardized experimental conditions [5, 6]. Moreover, adventitial inversion techniques have been widely adopted to control bleeding and prevent the formation of false lumens at the anastomotic site, and numerous studies have described their application [7–17]. However, the mechanical resilience of anastomoses employing these reinforcement techniques has not been adequately studied.

This study assessed the maximum permissible load and rupture mechanism of the anastomotic site using four anastomotic and reinforcement techniques by simulating an anastomotic model and subjecting it to longitudinal aortic tension.

## Materials and methods

### Study aim and objectives

This study aimed to evaluate the mechanical strength and rupture characteristics of various anastomotic techniques used in aortic surgery under standardized ex vivo conditions. To achieve this aim, we constructed standardized anastomosis models using porcine aortic tissue and prosthetic grafts. Four distinct suturing techniques were employed, incorporating combinations of simple interrupted sutures, adventitial inversion, Teflon-felt reinforcement, and horizontal mattress sutures. Uniaxial tensile testing was performed to quantify the maximum tensile load each anastomotic method could withstand. Additionally, rupture patterns were analyzed to assess the structural integrity associated with each technique, and statistical analyses were conducted to identify significant differences in biomechanical performance among the groups.

### Test specimen

**Fig. 1:**
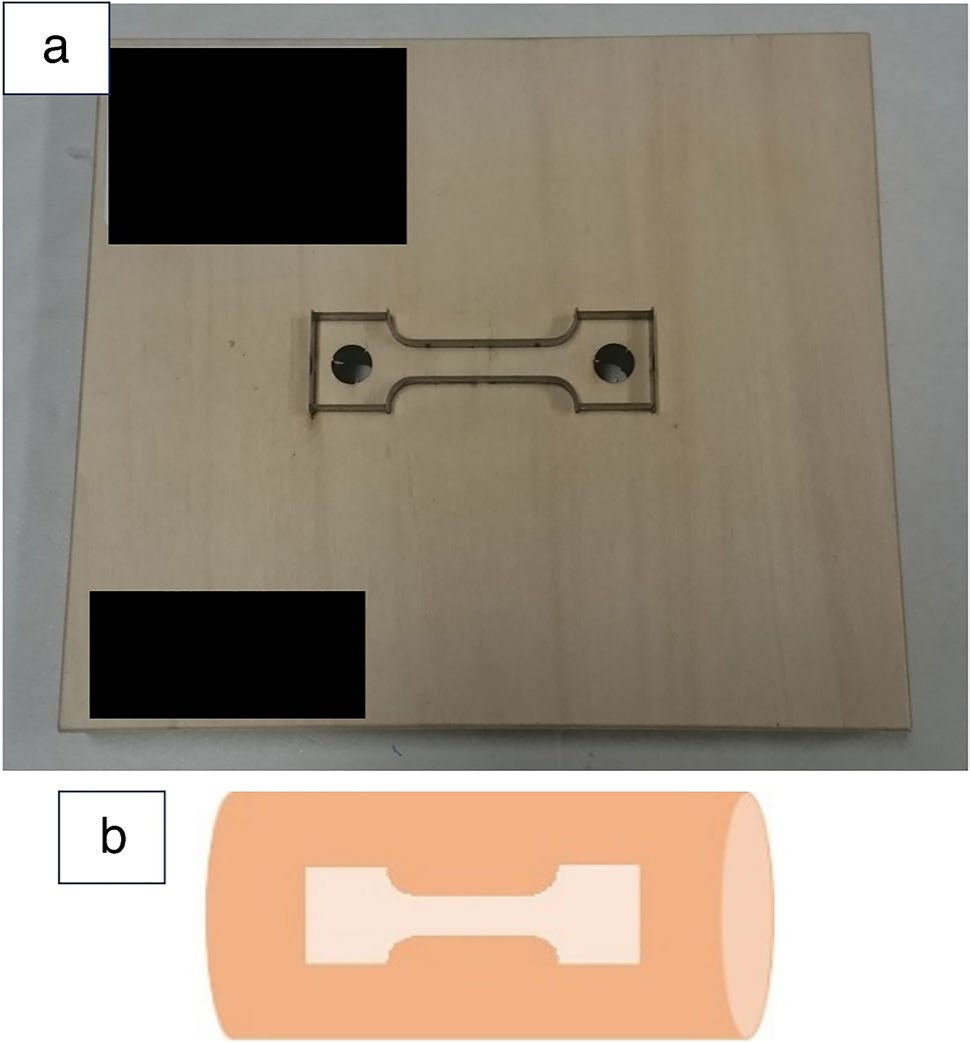
**a** Metal cutting dies. **b** Orientation of aortic wall sample

Uniaxial tensile tests were performed to characterize the mechanical behaviors of the anastomotic sites. We obtained fresh porcine aortae (*n* = 14) (Sankyo Labo Service Co., Inc., Tokyo) in accordance with institutional guidelines. The porcine aortae were cut open along the long axis. Dog bone–shaped specimens with a central zone (16 mm × 8 mm) were stamped from the opened-up aorta using metal cutting dies of appropriate dimensions (Fig. 1). A total of 56 specimens were prepared and randomly assigned to four groups (A, B, C, and D) in a completely random manner. In tensile testing, a dog bone–shaped specimen was utilized to ensure accurate and reproducible measurement of material properties. This geometry was specifically designed to minimize stress concentration at the clamping regions and ensure failure occurs within the central gauge section, where the material’s true mechanical properties could be evaluated. Prosthetic grafts (Triplex, Terumo, Tokyo, Japan) were cut into the same dog bone shape to ensure consistent comparisons during testing. These samples were subsequently bisected, facilitating anastomosis of the aortic wall to the prosthetic graft using the abovementioned techniques.

### Suture techniques

#### **Simple interrupted suture (Group A)**

**Fig. 2:**
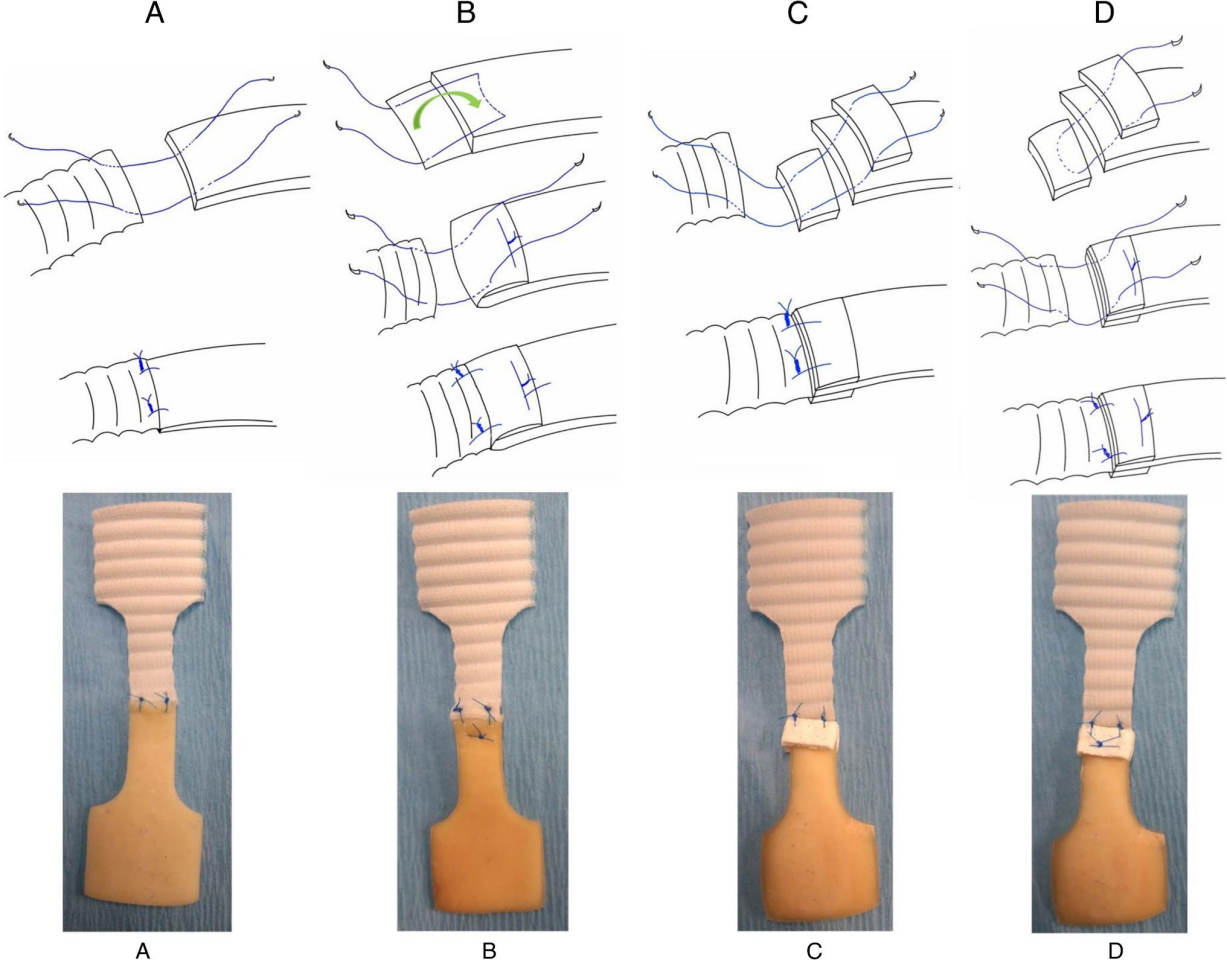
The actual anastomosis procedures and corresponding test specimens for Groups **A**, **B**, **C**, and **D**

The aorta and prosthetic graft were anastomosed using a 5–0 polypropylene monofilament suture. Two interrupted sutures were inserted 2 mm medial to the end and lateral edges. The distance between the two sutures was 4 mm (Fig. 2A).

#### Simple interrupted suture using adventitial inversion with a horizontal mattress suture (Group B)

The intima-media was removed and the left adventitia was trimmed to 5 mm beyond the level of the intimal edge of the central part of the halved specimen of the aorta, and the redundant adventitia was inverted into the aortic lumen and tacked to the luminal surface of the intima using a horizontal 5–0 polypropylene monofilament mattress suture. Two interrupted sutures were then inserted in the same fashion as in Group A (Fig. 2B).

#### Simple interrupted suture using a Teflon-felt sandwich (TFS) without a horizontal mattress suture (Group C)

Teflon felt (5 mm × 8 mm) was placed outside the adventitia and inside the intima. Sandwich anastomosis was performed with two interrupted sutures in the same fashion as in Group A (Fig. 2C).

#### Simple interrupted suture using a TFS with a horizontal mattress suture (Group D)

**Fig. 3:**
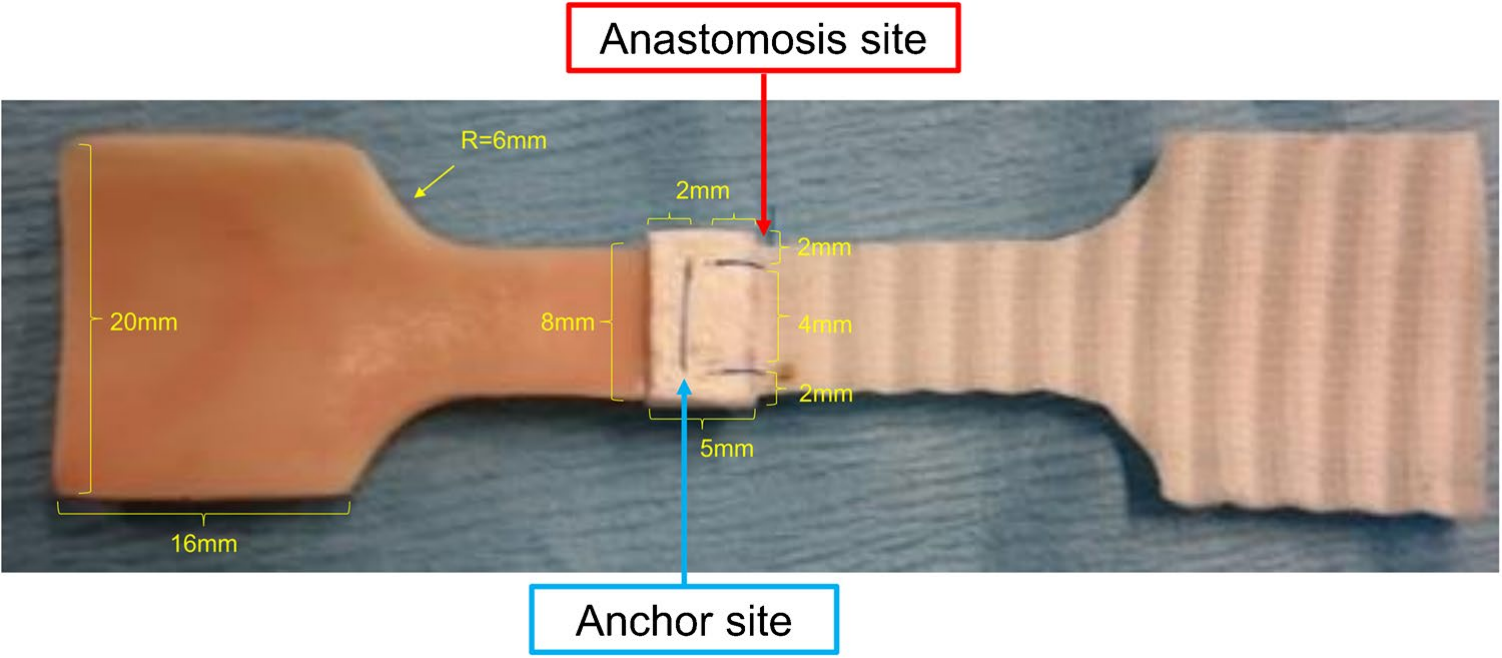
Nomenclature of the constructed test specimen components

Horizontal mattress sutures were added to secure the Teflon felts 3 mm medial to the end edge and 2 mm medial to the lateral edge, with a suture width of 4 mm. Sandwich anastomosis with two interrupted sutures was performed in the same manner as in Group C (Fig. 2D). For clarity, in this study, we referred to the sutured interface between the prosthetic graft and the porcine aorta as the “anastomosis site,” whereas the area where the TFS was secured to the porcine vessel wall was defined as the “anchor site” (Fig. 3).

While simple interrupted and continuous sutures differ structurally, in tensile testing of aortic-prosthetic graft anastomoses, the primary load-bearing points are at the suture sites on the aortic wall. Given this, tensile testing was performed using simple interrupted sutures to ensure consistent and controlled load application. In this study, to conduct tensile testing on the aortic wall, the specimens were shaped into a dog bone configuration to ensure that the load was applied to the center of the biological tissue. Given this setup, regardless of whether continuous or simple interrupted sutures were used, the primary load-bearing points remained the same. Therefore, we opted to perform tensile testing using simple interrupted sutures.

The thickness of each specimen was measured using a Mitutoyo micrometer (Mitutoyo Corporation, Japan), which can measure up to a 0.001 mm thickness. Each specimen’s thickness was measured five times. The average value of five measurements was used as the thickness of the specimen, and the deviation was approximately 0.01 mm.

### Experimental setup

Tensile assessments were executed using Instron® 4400 (Instron, Norwood, MA, USA). The apparatus was equipped with a specialized chuck (16 mm × 20 mm) tailored to the specimen’s dimensions, ensuring precise and consistent tests. The gripped part of the specimen was formed to fit the chuck shape. The specimens were then pulled in opposite directions at a speed of 5 mm/min, with this rate remaining constant and unchanged. The Instron® 4400 tensile test was performed at a constant crosshead speed of 5 mm/min, with load measurements recorded every 0.01 s. To ensure that no initial tension was applied to the specimen during setup, the test began from a slightly relaxed state, causing the initial load values to appear negative. To accurately determine the starting point of the measurement, a moving average of the load was calculated over 100 data points before and after each time step. The measurement officially commenced from the point where this moving average reached zero. The load was continuously applied rather than increased in discrete steps.

The specimens were constantly hydrated with saline to retain moisture. Given the wet state of the specimens, an anti-slip chuck was vital to secure them during evaluation. The consequent events, fracture or suture untying, were meticulously recorded. The peak load at the time of suture failure was also documented.

A comprehensive ex vivo assessment was conducted to evaluate the tensile strength resilience of the four suturing techniques: simple interrupted suture (Group A), simple interrupted suture using adventitial inversion with horizontal mattress suture (Group B), simple interrupted suture using TFS without a horizontal mattress suture (Group C), and simple interrupted suture using a TFS with a horizontal mattress suture (Group D); 5–0 polypropylene was used as the suture material in all the groups. Each technique was evaluated using 14 samples.

### Statistical analyses

A total of 56 specimens were evaluated. Statistical analyses were performed using R version 4.3.2. Continuous variables are presented as the median and interquartile range because of non-normally distributed data (the Shapiro–Wilk test). Therefore, non-parametric methods were employed. Differences among the four groups were analyzed using the Kruskal–Wallis test, followed by post hoc analysis with Dunn’s test using Bonferroni correction for multiple comparisons. Statistical significance was defined as *P* < 0.05.

## Results

### Aortic specimen thickness across the study groups

**Fig. 4:**
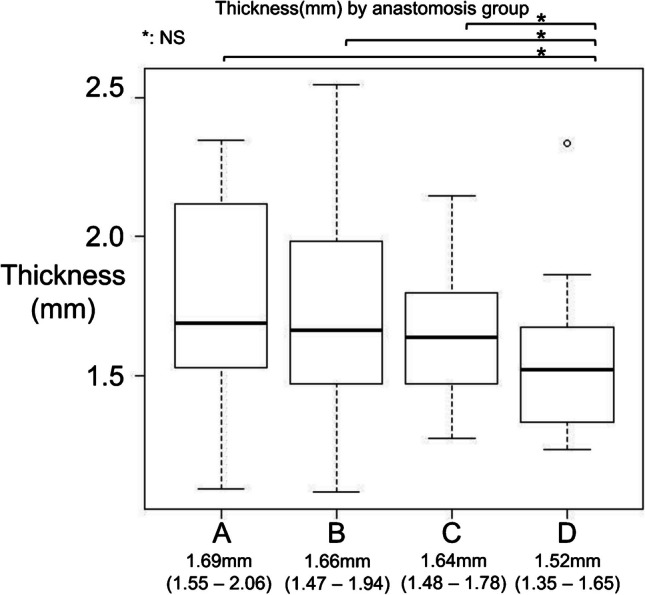
Thickness by anastomosis group. Boxplots showing the specimen thickness across the four anastomosis groups (**A**–**D**). Each box represents the interquartile range (IQR), with the horizontal line indicating the median. Median values and IQRs are shown beneath each group. No statistically significant differences were found among the groups (Kruskal–Wallis test, *P* = 0.413)

Our study commenced with a comparative analysis of the aortic specimen thickness across the four groups. Continuous variables are presented as the median and interquartile range (IQR). The relatively uniform thickness measurements underscored the consistency of sample preparation and selection. The median thickness was 1.69 mm (1.55–2.06) in Group A, 1.66 mm (1.47–1.94) in Group B, 1.64 mm (1.48–1.78) in Group C, and 1.52 mm (1.35–1.65) in Group D, as illustrated in Fig. 4. Statistical analysis indicated no significant variation among the groups (*P* = 0.413).

### Differential tensile strength in suture techniques

**Fig. 5:**
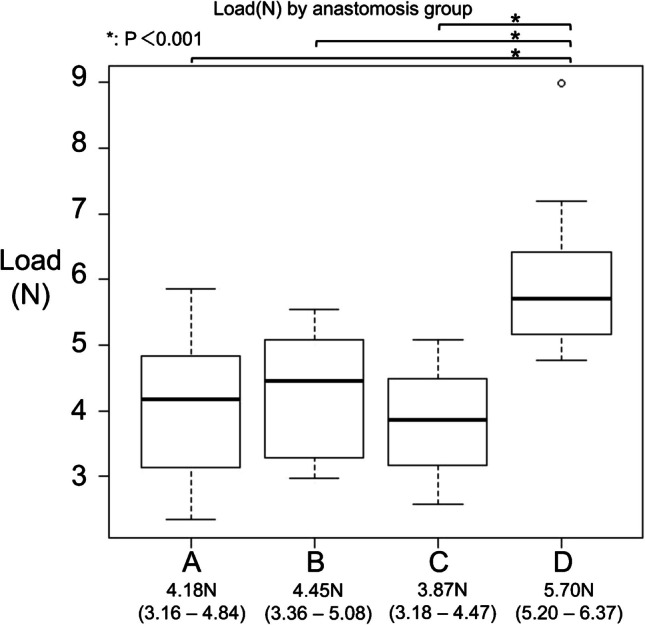
Load by anastomosis group. Boxplots showing the maximum load across the four anastomosis groups (**A**–**D**). Each box represents the interquartile range (IQR), with the horizontal line indicating the median. Individual values are shown as scattered dots. Median values and IQRs are displayed below each group. Statistically significant differences were observed between Groups **A** and **D**, **B** and **D**, and **C** and **D** (*P* < 0.001). *P*-values were calculated using the Kruskal–Wallis test, followed by Dunn’s test with Bonferroni correction for multiple comparisons. *Statistically significant

Our findings revealed a marked variation in tensile strength among the different suture techniques. As shown in the central figure, the load–displacement curves exhibited a qualitatively similar shape across all groups. Groups A and B demonstrated comparable elongation and tensile strength at the breaking point, with median maximum loads of 4.18 N (3.16–4.84) and 4.45 N (3.36–5.08), respectively. Group C showed a similar tensile strength to Groups A and B, with a median maximum load of 3.87 N (3.18–4.47), while exhibiting slightly greater elongation. The highest maximum load and elongation were observed in Group D, which exhibited a median maximum load of 5.70 N (5.20–6.37). Figure 5 highlights the marked resilience of Group D’s anastomosis technique, with a tensile force approaching 6 N. In contrast, Groups A, B, and C had median tensile strengths of approximately 4 N. Pairwise comparisons (Dunn’s test with Bonferroni correction) between Group D and the other groups yielded statistically significant differences (*P* < 0.01), reinforcing the superior tensile strength of Group D’s suture technique, which exhibited approximately 1.4 times the tensile force of the other methods.

### In-depth analysis of rupture patterns

**Fig. 6:**
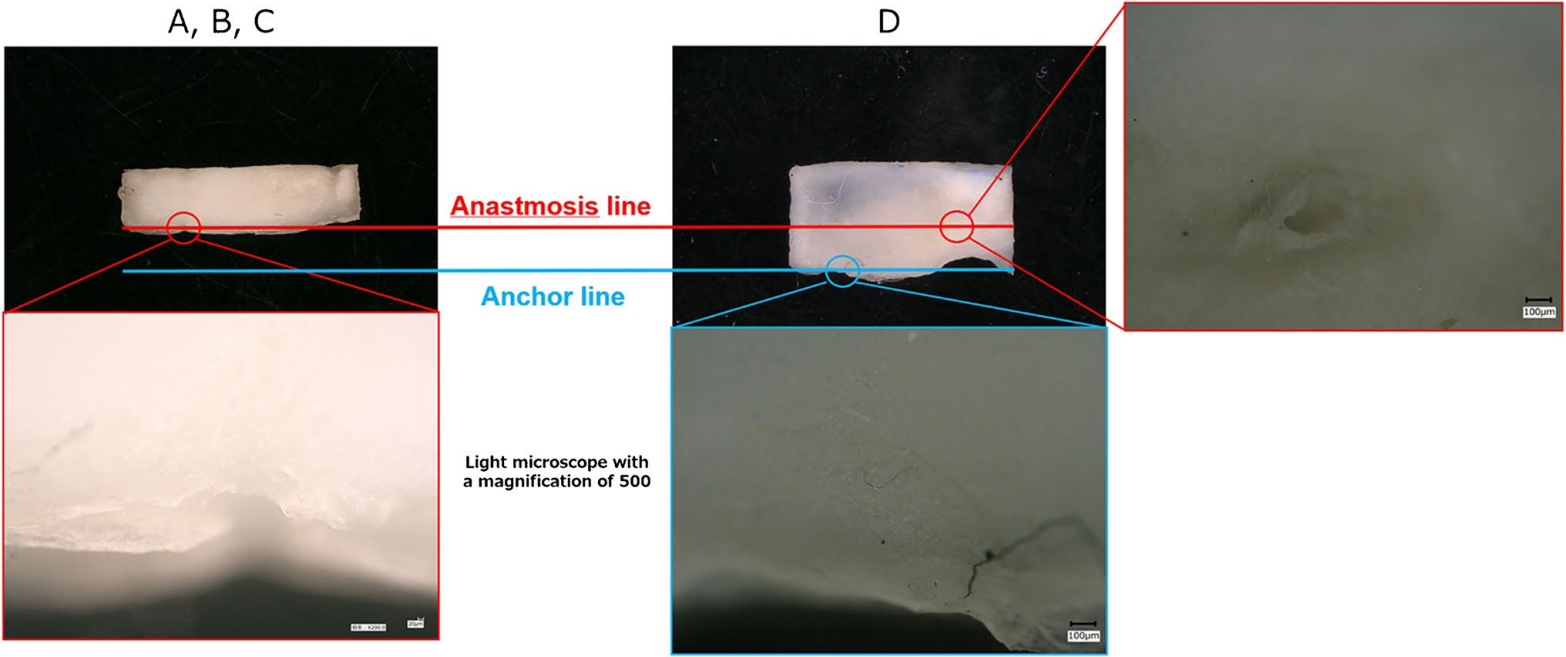
Rupture occurred at the interrupted suture line in Groups **A**, **B**, and **C**. Rupture occurred on the horizontal mattress suture line, and the interrupted sutures were preserved in Group **D**. Bar = 100 μm

**Fig. 7:**
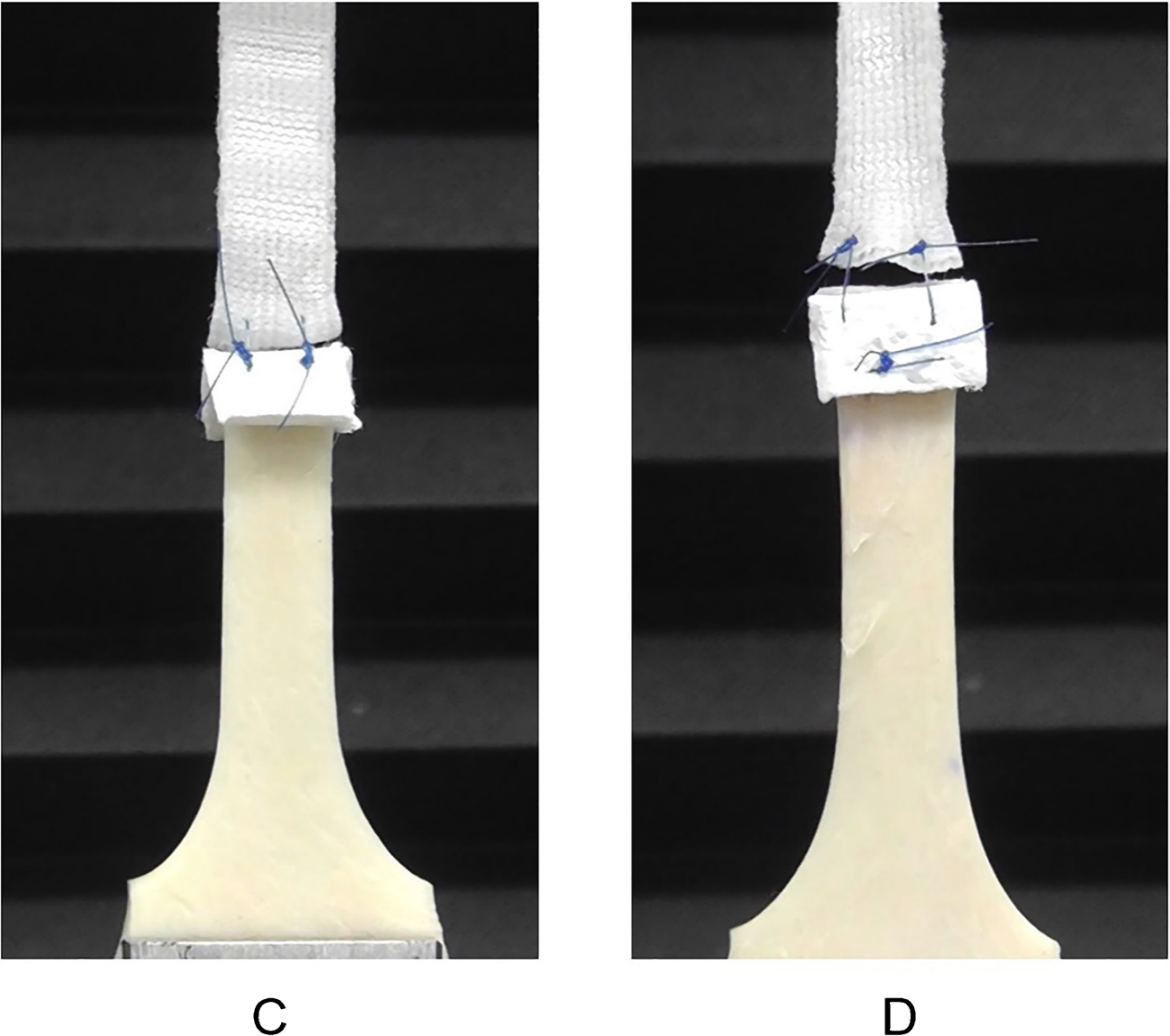
Differences in the felt behavior in Groups C and D during the tensile tension test

Tensile testing revealed distinct rupture patterns in the specimens, which were meticulously classified. In Group A, rupture predominantly occurred at the aortic anastomosis, which is referred to as the “anchor line” in this study. In Group B, rupture was observed at the anastomosis between the prosthetic graft and the aorta, similar to Group A; however, the horizontal mattress suture used for adventitial inversion remained intact. In Group C, rupture was localized along the interrupted suture line, with noticeable floating of the Teflon felt on the side opposite to the interrupted suture. In Group D, rupture occurred along the horizontal mattress suture line used to secure the Teflon felt. Notably, the interrupted suture hole remained intact, indicating the robustness of this suturing technique (Figs. 6 and 7).

## Discussion

Aortic anastomosis is critical in cardiovascular surgery, and the adopted methodologies significantly impact patient prognosis. We investigated this complex relationship using exhaustive research and revealed the biomechanical attributes of different suture techniques applied to aortic samples. Given the uniform thickness of the aortic samples across groups, the observed differences in tensile robustness and rupture patterns were particularly noteworthy.

A critical observation was the difference in rupture patterns across the groups (Fig. 6). Horizontal tearing from the interrupted suture hole in Groups A and B indicates that the tensile force added by the polypropylene monofilament suture is a probable factor in localized tissue damage. However, Groups C and D showed that additional components, such as Teflon felt, had a significant influence on the structural integrity of the tissue. Notably, Teflon felt, an industrial product renowned for its uniformity, revealed unexpected dynamics in the anastomosis process. Owing to its reduced elasticity compared with that of biological soft tissues, suture tension was applied directly to the Teflon, leading to potential rupture sites at the felt suture line.

Various reinforcement techniques have been introduced in aortic surgery to improve anastomotic stability and prevent complications. Although these methods are widely used in clinical practice, their mechanical performance under controlled conditions has not been fully elucidated. Floten proposed an adventitial inversion technique that provided a safe and secure anastomosis [7]. In clinical settings, techniques involving Teflon-felt reinforcement and adventitial inversion have been commonly applied to strengthen the aortic wall during reconstruction. While these approaches are often employed in aortic dissection surgery, their mechanical performance has not been systematically evaluated in isolation. The present study provides fundamental biomechanical data on these reinforcement methods using normal porcine aortic tissue under controlled ex vivo conditions. Tamura introduced a “turn-up” technique that involved the eversion of the graft ends, resulting in reduced bleeding and satisfactory clinical outcomes [18]. Ohata described a modified sandwich technique, using adventitial inversion and reinforcement with a felt strip, which ensures complete hemostasis [19]. Kan described a double telescopic anastomosis with an interrupted suture technique that minimized damage to the aortic wall and promoted anastomotic sealing [20]. Rylski presented an adventitial inversion with graft telescopic insertion technique, which ensured complete coverage of the inverted adventitia and achieved hemostasis [21]. Overall, these papers offered various anastomotic techniques for acute aortic dissection, each with advantages in reducing bleeding, promoting sealing, and achieving hemostasis.

Numerous studies have been conducted on the mechanical properties of aortic tissue, revealing their nonlinear and viscoelastic stress–strain relationship [22–24]. However, there is a lack of research on the mechanical behavior of the anastomotic site of the aorta using anastomotic techniques. The tensile strength of the anastomosis is a critical aspect that determines its capacity to expand to the point of rupture. If rupture occurs, the established tensile strength can provide insights into the longitudinal strength of the anastomosis.

The variability in tensile strength highlights the crucial role of suture methodology in determining anastomosis longevity. Our study emphasized that these techniques vary significantly, with certain methods displaying clear biomechanical superiority. Clinically, the method employed in Group D, which exhibited both tensile robustness and diminished susceptibility at the segmented suture locations, is a potent option for intricate aortic interventions. Considering the continuous physiological pressure faced by aortic grafts, this heightened durability is likely to be associated with better patient outcomes.

Additionally, the cataloged rupture patterns elucidate the potential weaknesses of each suture method. These insights hold promise for refining current techniques, mitigating their vulnerabilities, and potentially ushering in a new era of safer and more reliable cardiovascular surgery. Our ex vivo study showed that the TFS technique resisted a significantly higher tension than the other tested methods. These ex vivo findings suggest that this technique may offer mechanical advantages in aortic anastomosis. However, the study was conducted using normal porcine aortic tissue under controlled laboratory conditions, and the results may not directly translate to clinical settings.

In studying the mechanical properties of biological soft tissues such as the aorta, it is essential to consider their nonlinear and anisotropic characteristics, which arise from their material composition and structural organization [24]. The aorta, for instance, consists of three layers—intima, tunica media, and adventitia—where collagen, elastin, and smooth muscle fibers are primarily oriented circumferentially. This structural anisotropy significantly influences the tissue’s mechanical behavior. Ideally, tensile testing should incorporate the anisotropy of the entire tissue, including the circumferential direction.

Multiple studies have reported that the aorta exhibits greater strength in the circumferential direction compared to the longitudinal direction [25–29]. Despite these findings, the mechanical behavior of the longitudinal direction, which has been reported to exhibit relatively lower strength, has been less thoroughly investigated. To address this gap, our study focused on evaluating the mechanical response in the longitudinal direction to provide deeper insights into its biomechanical behavior.

### Limitations

It should be noted that our experiments were conducted ex vivo, excluding variables present in vivo, such as blood pressure oscillations, inflammatory responses, and interactions with surrounding tissues. Additionally, while our results provide valuable insights into the immediate biomechanical response, we did not account for long-term clinical outcomes such as graft infections, chronic inflammation, or other tissue responses that could influence patient prognosis. Lastly, factors such as surgical expertise, patient-specific anatomical and physiological differences, and postoperative care, which are critical in shaping surgical outcomes, were beyond the scope of this study.

## Conclusions

The TFS technique had greater tensile strength than that of the simple interrupted suture, simple interrupted suture using adventitial inversion with horizontal mattress sutures, or simple interrupted suture using a TFS without a horizontal mattress suture. This finding can serve as a valuable technical foundation and warrants experimental assessment in vivo to assess its merits in a clinical setting.

## Supplementary Information

Below is the link to the electronic supplementary material.

Supplementary file1 (MP4 15 MB)

## Data Availability

The data underlying this article will be shared on reasonable request to the corresponding author.
